# The effect of adding curcumin to sodium valproate in treatment of patients with bipolar disorder in the acute phase of mania: A randomized double-blind clinical trial

**DOI:** 10.3389/fpsyt.2023.1068277

**Published:** 2023-02-02

**Authors:** Farzad Akbarzadeh, Nabahat Niksun, Ghazal Behravan, Fatemeh Behdani, Amir Hooshang Mohammadpour, Mahmoudreza Jaafari, Tayebeh Hosseini, Zahra Rajai, Alireza Ebrahimi, Mahboubeh Eslamzadeh

**Affiliations:** ^1^Psychiatry and Behavioral Sciences Research Center, Faculty of Medicine, Mashhad University of Medical Sciences, Mashhad, Iran; ^2^Department of Clinical Pharmacy, Mashhad University of Medical Sciences, Mashhad, Iran

**Keywords:** curcumin, clinical trial, sodium valporate, bipolar disoder, bipolar disorder type 1

## Abstract

**Background:**

Inflammatory processes play a role in the etiopathogenesis of bipolar disorder type 1. Full therapeutic responses are seldom seen and the ongoing inflammatory processes in the brain could lead to neuronal loss. Curcumin, a relatively safe herbal compound, has been shown to have anti-inflammatory effects. The present randomized double-blind clinical trial study aimed to investigate the effect of adding curcumin to the treatment regimen of BID.

**Materials and methods:**

This randomized double-blind clinical trial was conducted on 78 patients diagnosed with BID according to the Diagnostic and Statistical Manual of Mental Disorders (DSM 5) criteria. The sample were divided into two groups. Patients in both groups received sodium valproate starting at a dose of 600 milligrams per day and administered up to 20 milligrams per kilogram per day or the highest dosage of the patient’s tolerance. Patients in the intervention group also received curcumin as nanomicelle in soft gelatin capsules 40 milligrams per day. The control group received placebo tablets with the same characteristics as the curcumin tablets. They were assessed by a psychiatrist using the Young Mania Rating Scale (YMRS), Mini-Mental State Examination (MMSE), Clinical Global Impression (CGI), and a medication side effect questionnaire at the beginning of the study, as well as in the first, second, and fourth weeks of the study.

**Results:**

Among the 78 patients chosen to participate in the project, 54 people completed the trial. No specific side effect was observed in the two groups. Both groups showed an increase in their MMSE scores compared to the beginning of the study (value of *p* < 0.001). Although this increase was not statistically different between the two groups (value of *p* = 0.68). The YMRS score of both groups decreased significantly by the end of the study (value of *p* < 0.001); however, this decrease was not significantly different between the two groups (value of *p* = 0.64). In addition, the two groups experienced a significant increase in their CGI scores throughout the study (value of *p* < 0.001), this increase however was not statistically different between the two groups (value of *p* = 0.88).

**Conclusion:**

The present study suggested that curcumin may not be a useful adjuvant agent in the management of patients with BID receiving sodium valproate as treatment.

Clinical trial registration: Iranian Registry of Clinical Trials (IRCT), identifier IRCT2016102530504N1.

## Introduction

Bipolar I disorder (BID) is a life-long episodic disease characterized by changes in a person’s mood varying between mania and depression. During mania episodes, effective and timely diagnostic and therapeutic interventions are required to minimize the disorder’s harmful effects on the individual as well as the interpersonal side effects which could easily interrupt the affected person’s life (1). Therefore, investigating an effective medical treatment preserving various dimensions of the personal and social life of patients is the main focus of research. Psychopharmacological treatments are the first-line treatment. So far, mood stabilizers including carbamazepine, sodium valproate, and some antipsychotics have been used to treat the acute phase of mania (1). The necessity of performing more precise investigations to find new therapeutic approaches becomes more apparent by considering bipolar disorder’s prevalence (1.5%), suicidal rate (10–20%), considerable recurrence, and the half effectiveness of single-drug therapy, as well as inefficient therapeutic interventions including drug augmentation for managing cases who show therapeutic resistance (1). The direct and indirect costs imposed on patients’ families as well as society during the acute phase of the disease is much greater than the cost required for the proper and timely treatment of the disease (1). Although the underlying molecular mechanisms for BID are not fully understood, most available drugs regulate the abnormal level of specific biochemical factors. Curcumin, a relatively safe herbal compound, has been long used for the treatment of various diseases and has been suggested as a potential therapeutic option for psychiatric illnesses. Among various underlying mechanisms for the development of BID, some studies demonstrated that abnormal serum levels of brain-derived neurotrophic factor (BDNF) are seen in BID patients (2). Therefore, some of the main therapeutic drug regimens increase the BDNF levels while reducing BID symptoms. Similar to well-known treatments (including lithium and atypical antipsychotics), curcumin can increase BDNF levels in BID patients (3–5). Alongside the abnormal level of BDNF in BID patients, there is a growing line of evidence suggesting that oxidative stress mechanisms are involved in BID (6, 7). In this way, increased reactive oxygen species (ROS) production results in apoptosis of brain cells (6–11). Moreover, inflammatory processes affect neurotransmitter function and neuronal plasticity (12, 13), which play a part in the pathophysiology of neurodegenerative diseases (14). An increase in inflammatory markers including C-reactive protein (CRP), inflammatory cytokines, and tumor necrosis factor (TNF) alpha is seen in BID (15). Studies have shown that curcumin has antioxidant functions that can reduce ROS, regulate inflammatory responses and decrease apoptosis (16–19).

**Figure 1:**
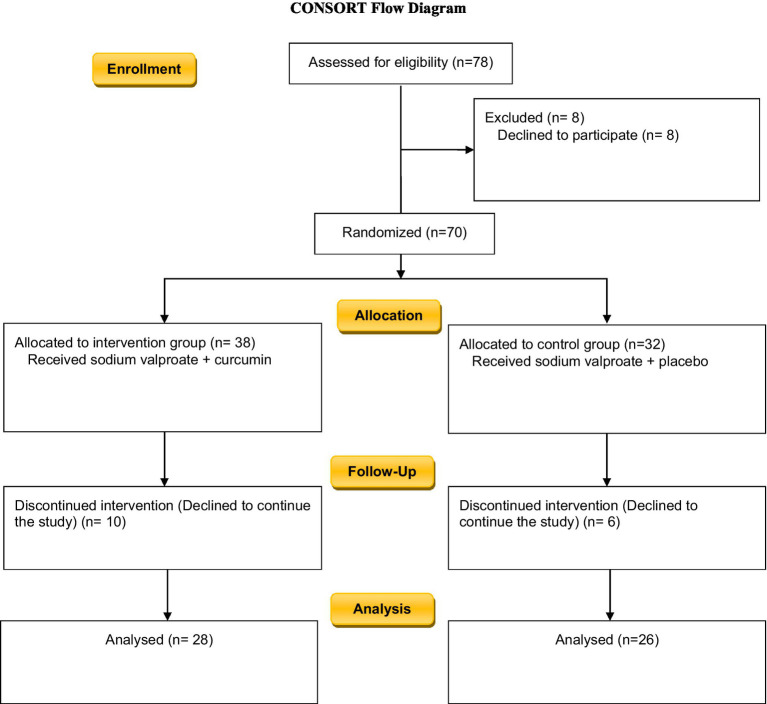
Study flow chart.

**Figure 2:**
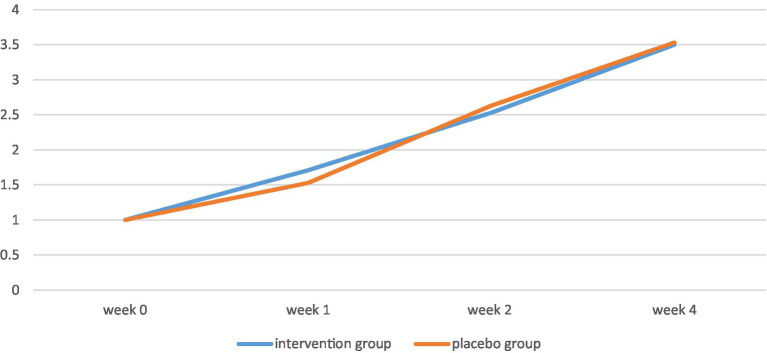
CGI scores of the two groups throughout the study.

**Figure 3:**
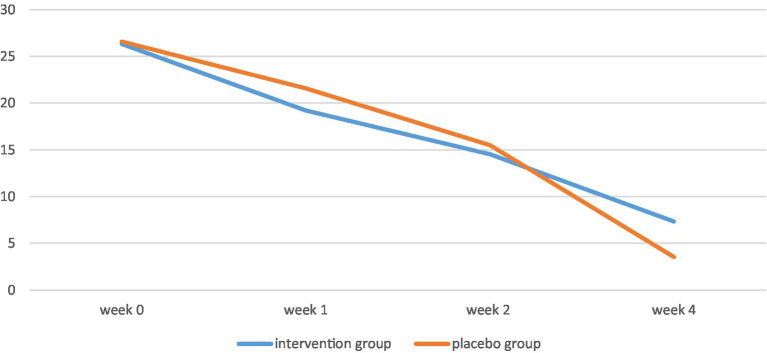
YMRS scores of the two groups throughout the study.

Since the use of curcumin has been long considered as a potent herbal drug in ancient Persian medical texts, many studies have been carried out on this subject but limited studies are evaluating its effects on psychiatric disorders including BID. The present study aimed to determine the effect of adding curcumin to the therapeutic regimen of patients with BID during the acute phase of mania (Figures 1–3).

## Materials and methods

The present double-blind randomized clinical trial study approved by Mashhad University of Medical Sciences was conducted at Ibn-Sina Psychiatric Hospital in Mashhad, Iran from 2016 to 2017. A total number of 78 patients diagnosed with BID based on the Diagnostic and Statistical Manual of Mental Disorders 5, participated in the study.

### Inclusion criteria

The inclusion criteria were as follows:

- Diagnosis of bipolar-1 disorder with recent mania episode based on DSM-5 diagnostic criteria by semi-structured interview by a psychiatrist and diagnostic confirmation with a score of at least 20 on the Young Mania Rating Scale (YMRS) (20).
- The absence of the diagnosis of schizophrenia, delirium, bulimia nervosa, anorexia nervosa, intellectual disability, autism, and attention deficit hyperactivity disorder (ADHD).
- Age between 18 and 50.
- No substance abuse in the last 3 months.
- No history of admission to psychiatric wards during the past 3 months.
- No history of seizure or epilepsy.
- **T**he absence of suicidal or homicidal risk during the psychiatric interview.
- Not being pregnant.
- Lack of a history of susceptibility to sodium valproate or any herbal medicines.
- Patients who had received other medications during the last 2 weeks before or during the study (including mood stabilizer treatments) underwent a washout period based on the medication’s half-life for patients receiving drugs with a long half-life.

A full interview was conducted for each patient by a psychiatric resident and informed consent was obtained from eligible patients or their legal guardians. All participants were assured that their information will be kept confidential, and that they would be able to leave the study at any time. Furthermore, patients who needed urgent intervention or were suspected of intensifying symptoms by the treatment were excluded from the study.

### Medications and questionnaires

Patients who agreed to participate in our trial were randomly assigned into two groups using a randomized number table. Patients in the intervention group received sodium valproate (starting at a dose of 600 mg/day and administrated up to 20 mg/kg/day or the highest dosage of the patient’s tolerance) and curcumin as nanomicelle in soft gelatin capsules (Sinacurcumin®), 40 mg once daily. The oral bioavailability of curcumin is very low due to the hydrophobic nature of this compound. Kunnumakkara et al. (21) to improve the oral bioavailability of curcumin, we used SinaCurcumin oral capsules (Exir Nano, Tehran, Iran) in this study. SinaCurcumin is a registered product from curcuminoids in Iran (IRC: 1228225765). Curcuminoids are dietary polyphenols extracted from the dried rhizomes of Curcuma longa L (turmeric) and comprise curcumin, desmethoxycurcumin, and bisdemethoxycurcumin, which are together known as the C3 complex. Each soft gelatin capsule of SinaCurcumin contains 40 mg curcuminoids as nanomicelles, absorbed as equivalent to 500 mg curcumin tablets. The encapsulation efficiency of curcuminoids in nanomicelles is almost 100%. The mean diameter of nanomicelles is around 10 nm. The oral absorption of SinaCurcumin is at least 50 times more than the conventional powder of curcumin in mice (22). The control group received a placebo in addition to sodium valproate. Placebo soft gels were prepared by the same company, in the exact same appearance as the curcumin tablets, containing all ingredients of the other soft gel except curcumin, with the same dosing.

The Young Mania Rating Scale (YMRS) is a rating scale that is traditionally used to scale symptoms of mania; it is used by clinicians and psychiatrists and typically takes 15–30 min to complete. It consists of 11 items that require the patient to subjectively report their symptoms in the past 48 h. Its validity and reliability has been proven for the Iranian population (23). Clinical Global Impression (CGI) rating scales are used to measure the severity of mental disorders as well as their response to treatment. The CGI is used by clinicians and consists of three measures: severity of illness, global improvement, and efficacy index. The validity and reliability of CGI has been proven. It is rated on a 7-point scale, 1 representing normality and 7 representing “among the most severely ill patients” (24). The Mini-Mental State Examination (MMSE) is a 30-item questionnaire used to assess different aspects of cognition including orientation, memory, attention, recall, and registration. It is widely used to screen for dementia. A person can earn a maximum score of 30 from this questionnaire. The score 24 is considered as the standard cut-off for distinguishing normal from impaired cognition. The validity and reliability of this questionnaire has been approved for the Persian population (25).

### Evaluations

Participants in both groups were evaluated at baseline, week one, week two, and week four of the study after receiving the assigned drug regimens using the clinical global impression (CGI) and YMRS (20, 26, 27). In addition, a self-report medication side effect questionnaire was used to investigate the possible side effects of the used medicine in the studied groups. Mini-mental state examination (MMSE) was performed at the end of the first and fourth weeks of treatment.

It should be noted that due to the lipophilic nature of curcumin, the oral absorption of curcumin in common oral forms (powder, capsule, and pill) may not be sufficient. In the present study, soft gelatin capsules containing curcumin nanomicelles (SinaCurcumin, Tehran, Iran) were used. The curcumin in the soft gel is blocked in the hydrophobic section of curcumin nannies. These spherical nanomicelle particles have a size of about 10 nm and increase the water solubility of curcumin. After oral administration, soft gel capsules containing curcumin nannies disintegrate in the acidic environment of the stomach in less than 15 min. These nanomicelles remain there for at least 6 h before they get to the small intestine intact.

Upon reaching the small intestine, nanomicelles facilitate the transfer of curcumin from the inert surficial water layer of the intestinal epithelial cells, which inhibits the absorption of lipid-soluble compounds and increases the oral absorption of curcumin (Calculation of absorbed curcumin).

The possible side effects of the medications were evaluated by psychiatric residents in each visit using a checklist, and if necessary, the essential laboratory tests were ordered under the supervision of a psychiatrist.

Since there were no similar studies, the sample size could not be calculated using statistical formulas. Therefore, this study was done as a preliminary study with a sample size of at least 25 people in each group.

Descriptive statistics, including frequency tables, diagrams, and statistical indicators were used, and data analysis was done using SPSS software version 11.5. Kolmogorov–Smirnov test, Mann Whitney test, independent *t*-test, Fisher’s exact test, and chi-square were applied and a value of *p* of less than 0.05 was taken as mean statistical significance. To control and determine the effect of confounding variables in case of heterogeneity between the two groups, appropriate statistical tests including covariance or logistic regression were used.

## Results

Among the 78 individuals who agreed to take part in this study, 8 individuals discontinued sodium valproate, two patients changed their therapeutic regime as their diagnosis was changed, 8 persons refused to participate in the study and 4 cases were excluded because of their lack of cooperation. A total number of 54 individuals continued **t**he study until the end.

The mean age of participants in the intervention group and control group was 36.28 ± 10.73 and 32.42 ± 9.60, respectively (*p* = 0.172). Most of the patients in the intervention group were male (23 versus 12 individuals) while most of the patients in the control group were female (9 versus 6 patients; *p* = 0.091). The patients’ demographic data is summarized in Table 1.

**Table 1:** Demographical information of the studied population.

	Placebo group	Curcumin group	Value of *p*
Gender			
Male	6 (40.0%)	23 (65.7%)	
Female	9 (60.0%)	12 (34.3%)	
Age	32.42 ± 9.6	36.28 ± 10.73	0.17*
Age of onset of disease	25.57 ± 7.48	25.92 ± 7.01	0.85*
Job			
Homemaker	7 (26.9%)	12 (42.9%)	0.69**
Unemployed	16 (61.5%)	11 (39.3%)	
Retired	1 (3.8%)	1 (3.6%)	
Self-employed	1 (3.8%)	2 (7.1%)	
Full-time student	1 (3.8%)	2 (7.1%)	
Education			
Primary school	11 (42.3%)	16 (57.1%)	0.56**
High school diploma	12 (46.2%)	10 (35.7%)	
Higher than high school diploma	3 (11.5%)	2 (7.1%)	
Number of previous hospitalizations			
1 or less	10 (38.5%)	5 (17.9%)	0.17***
2–3 times	13 (50.0%)	16 (57.1%)	
More than 3 times	3 (11.5%)	7 (25.0%)	
History of inflammatory disease			
Yes	2 (7.7%)	3 (10.7%)	>0.99**
No	24 (92.3%)	25 (89.3%)	
Family history of depression			
Yes	15 (57.7%)	10 (35.7%)	0.1***
No	11 (42.3%)	18 (64.3%)	
History of depression			
Yes	10 (38.5%)	6 (21.4%)	0.17***
No	16 (61.5%)	22 (78.6%)	
History of physical illness			
Yes	8 (30.8%)	10 (35.7%)	0.77**
No	18 (69.2%)	18 (64.3%)	
Medicine			0.06**
Risperidone	9 (34.6%)	10 (35.7%)	
Alprazoplam	2 (7.7%)	8 (28.6%)	
Olanzapin	8 (30.8%)	4 (14.3%)	
Lithium	2 (7.7%)	5 (17.9%)	
Tranqopine	5 (19.2%)	1 (3–6%)	

*Independent *t*-test. **Fisher’s exact test. ***Chi-squared test.

No side effect was seen in any of the two groups. There was no significant difference between the two groups in terms of the MMSE score obtained on week 0 (value of *p* = 0.16), and week 4 (value of *p* = 0.17) of the study. However, in both groups, significantly higher MMSE scores were achieved by the end of the study compared to week 0.

The YMRS score significantly decreased in both the intervention group and the control group from week 0 to week 4 (value of *p* < 0.001). There was no statistically significant difference among the YMRS score of the two groups in week 0 (value of *p* = 0.87), week 1 (value of *p* = 0.18), week 2 (value of *p* = 0.61), and week 4 (value of *p* = 0.71) of the study.

By the end of the study, CGI scores of both groups had increased significantly (value of *p* < 0.001). However, there was no significant difference between the CGI scores of the control and the intervention group in week 1 (value of *p* = 0.36), week 2 (value of *p* = 0.5), and week 4 (value of *p* = 0.88) of the project.

The MMSE score was significantly different in each group by week 4 of the study (*p* < 0.001; Table 1). To compare these changes between the two groups, Mann–Whitney test was used and no significant difference was observed (*p* = 0.68). Although the decrease in YMRS scores in each group was significant overtime (*p* < 0.001; Table 1). However, there was no significant difference among the study groups after 4 weeks (*p* = 0.64). Despite the significant increase in CGI scores in each group (p < 0.001; Table 1), there was no significant difference between the study groups after 4 weeks (*p* = 0.93).

While the percentage of response to treatment in the intervention group was higher than the control group, Fisher’s exact test did not confirm any significant difference between the two groups (*p* = 0.49; Table 2). In both groups, more than half of the patients reported subsidence of symptoms which was greater in the intervention group but was not statistically significant among study groups (*p* = 0.75; Table 2).

**Table 2:** Changes in MMSE scores of the two groups in 4 weeks.

MMSE	Curcumin group	Placebo group	Value of *p*
Week 0	22.42 ± 4.8	20.92 ± 4.15	0.16*
Week 4	24.6 ± 4.55	23.53 ± 3.78	0.17*
Changes from week 0 to week 4	2.17 ± 2.4	2.61 ± 2.88	0.68*
Value of *p*	<0.001**	<0.001**	

*Mann–Whitney. **Wilcoxon test.

## Discussion

The present clinical trial study demonstrated that after 4 weeks of addition of curcumin to sodium valproate in BID-1 patients, there was not a significant improvement in MMSE, YMRS or CGI in contrast to patients receiving valproate alone (Tables 3, 4).

**Table 3:** Changes in YMRS scores among the two groups in the study.

YMRS score	Curcumin group	Placebo group	Value of *p*
Week 0	26.32 ± 6.13	26.57 ± 5.19	0.87*
Week 1	19.21 ± 6.78	21.57 ± 6.11	0.18*
Week 2	14.53 ± 7.08	15.5 ± 6.88	0.61*
Week 4	7.32 ± 6.27	7.96 ± 6.58	0.71*
Value of *p*	<0.001**	<0.001**	

Dependent *t*-test. **Repeated measure.

**Table 4:** Changes in the CGI score of the two groups in the study.

CGI score	Curcumin group	Placebo group	Value of *p*
Week 0	1 ± 0	1 ± 0	–
Week 1	1.71 ± 0.65	1.53 ± 0.5	0.36*
Week 2	2.53 ± 0.63	2.63 ± 0.65	0.5*
Week 4	3.5 ± 0.83	3.53 ± 0.7	0.88*
Value of *p*	<0.001**	<0.001**	

*Mann–Whitney. **Friedman test.

BID is a life-long episodic disease characterized by changes in a person’s mood between mania and depression. During mania episodes, effective and timely diagnostic and therapeutic interventions are required to minimize side effects, which can easily interrupt an individual’s normal life. Many BID patients often experience recurrent episodes throughout their lives if they do not receive proper treatment and follow up. The acute phase of manic episodes can have unpleasant effects on the patients’ mental status and their family (1). Therefore, pharmaceutical treatment of patients’ symptoms is an area of interest for researchers. Psychopharmacological treatments are the first line for treating BID. So far, mood stabilizers, including carbamazepine, sodium valproate, as well as antipsychotics, have been used to treat the acute phase of mania (1). Incomplete response to existing drugs among considerable number of patients made investigation for novel and effective medicines a global concern.

Although the exact mechanism of development of BID is not clear; inflammation, cellular apoptosis and increased BDNF level are considered among the probable mechanisms for BID. Curcumin is an old herbal medication which is thought to be effective in lowering inflammatory status, controlling apoptosis and regulating BDNF level (28). Nowadays, the complimentary usage of herbal and ancient medicine in modern practice has become an area of interest for many researchers. Thus many researchers are looking into using ancient herbal medicine in combination with new pharmacological drugs to find an effective treatment for BID.

Part of the anti-inflammatory effects of turmeric may be due to the inhibitory effects of curcumin on the activity of the hyaluronidase enzyme. Moreover, curcumin inhibits the expression of COX2 and the production of cytokines such as interferon-gamma and interleukin-6 which results in inhibiting the inflammatory responses (29). Previous studies have claimed curcumin has anti-inflammatory effects in deactivating free radicals. Moreover, they have reported anticancer effects for curcumin. These effects of curcumin are often dose-dependent vary based on the environment (29–31). Brietzke et al. (32) evaluated curcumin’s anti-inflammatory and antioxidant properties and reported that this agent was notably affecting depressive symptoms and cognitive impairment. However, they suggested further clinical trials to support their findings. A study by Arora et al. (33) demonstrated that curcumin could reduce behavioral deficits and depression in animal models in a dose-dependent manner. They also suggested that curcumin can increase dopamine and serotonin levels while decreasing inflammatory markers (33). Choudhary et al. (34) demonstrated similar effect for curcumin on mice and reported that curcumin can improve memory impairment, depression and behavioral problems. A recent review on clinical trials evaluating the role of curcumin in depressive patients demonstrated that among clinical trials, 2 studies found that curcumin has antidepressant activity in contrast to placebo groups after 8 and 12 weeks duration (35). Moreover, a paper by Yu et al. (36) demonstrated that the addition of 1,000 mg of curcumin to the selective serotonin reuptake inhibitor (SSRI) could improve the antidepressant effects of the SSRIs.

The only available animal study about the efficacy of curcumin in bipolar disorder is on animal mania models receiving ketamine (37). The rats had ketamine-induced hyperlocomotion and oxidative damage in prefrontal cortex and hippocampus. The authors demonstrated that pretreatment with curcumin could prevent hyperlocomotion and reverse the oxidative stress parameters (37). Our study was the first clinical trial on human subjects using curcumin as a treatment for BID and could not confirm a therapeutic effect for curcumin as an adjuvant for valproate. The curcumin therapeutic effects appear to be because of their neuroprotective and antioxidant effects, which increase the level of neurotrophic factors. Given the low bioavailability of the drug, it seems necessary to increase the dose. Concerning other studies on non-BID patients, a dose of 500 mg per day, which was used in ours, also seems to be a low dose of curcumin (38–41). Kulkarni et al. (42) recommended to start treatment with a dose of 20 mg based on the patient’s weight and increase the dose to 80 mg per kg, which is about 1,500 mg per day to achieve the antidepressant effects of curcumin. Moreover, a four-week follow-up period may be limited and insufficient; and further studies with longer follow-ups may provide different results. It is suggested that future studies use higher doses of curcumin, as well as larger sample sizes and longer follow-up intervals to further investigate the possible effects of curcumin on the treatment of bipolar disorder. There are also other possible challenges that need to be considered regarding the addition of curcumin to the treatment regimen of different disorders, as further research is required to better investigate their possible efficacy as well as possible side effects on different body systems and probable interactions with other medications used by patients. It should also be noted that due to cultural reasons, it may cause a change in the patient’s adherence to medication.

## Limitations

The limited small sample size and the fixed dosage of the curcumin were our study’s constraints. For future studies, we suggest the use of higher doses of curcumin, which is recommended to be administrated according to the curcumin serum levels of participants.

## Conclusion

The present clinical trial study demonstrated that after 4 weeks of addition of curcumin to sodium valproate in BID patients, there was no significant improvement in MMSE, YMRS, or CGI in contrast to patients receiving valproate alone. Further clinical studies with higher doses, greater sample sizes and longer follow-up durations are needed.

## Data availability statement

The raw data supporting the conclusions of this article will be made available by the authors, without undue reservation.

## Ethics statement

The studies involving human participants were reviewed and approved by Mashhad University of Medical Sciences. The patients/participants provided their written informed consent to participate in this study.

## Author contributions

All authors listed have made a substantial, direct, and intellectual contribution to the work and approved it for publication.

## Conflict of interest

The authors declare that the research was conducted in the absence of any commercial or financial relationships that could be construed as a potential conflict of interest.

## Publisher’s note

All claims expressed in this article are solely those of the authors and do not necessarily represent those of their affiliated organizations, or those of the publisher, the editors and the reviewers. Any product that may be evaluated in this article, or claim that may be made by its manufacturer, is not guaranteed or endorsed by the publisher.
